# Measurement of the Time Required for a Termite to Pass Through Tunnels with Different Curvatures

**DOI:** 10.1673/031.012.6401

**Published:** 2012-05-20

**Authors:** Seungwoo Sim, Sang-Hee Lee

**Affiliations:** Division of Fusion Convergence of Mathematical Sciences, National Institute for Mathematical Sciences, Daejeon, South Korea

**Keywords:** foraging efficiency, termite tunnel curvature, termite tunnel network, traffic efficiency

## Abstract

The subterranean termite, Reticulitermes speratus kyushuensis (Isoptera: Rhinotermitidae), excavate complex tunnel networks below the ground for foraging. The tunnels are either curved or meandering. In our previous study, results showed that termites passed smooth—rounded corners faster than they did around sharp corners. Smooth—rounded corners can be mathematically quantified by the curvature, representing the amount by which a geometric object deviates from a straight line. The present study explored how the time spent inside a tunnel changes in accordance with the degree of tunnel curvature. To do so, artificial tunnels with different curvatures were constructed in acryl substrates. Tunnels were 5 cm in length with widths of *W* — 2, 3, or 4 mm, and the distance between the two ends of the tunnel was *D* = 2, 3, 4, or 5 cm. A higher value of *D* signified a lower curvature. The time (τ) taken by a termite to pass through the tunnel was measured. In the case *of W* = 2 mm, the values of τ were statistically equal for *D* = 2, 3, or 4 cm, while τ for *D* = 5 cm was significantly lesser. In the case of *W =* 3, τ was statistically more for *D* = 2 and 3 cm than it was for *D* = 4 and 5 cm. For *W =* 4, τ was statistically equal for *D* = 2 and 3 cm, while τ for *D* = 4 cm was relatively shorter. Interestingly, the value of τ when *D* = 5 cm was statistically the same as *D* = 3 or 4 cm. These resulted from two types of termite behavior: biased walking and zigzag walking.

## Introduction

Subterranean termites forage by constructing tunnel networks below the ground (Su et al. 1984). The network geometry reflects a compromise between foraging efficiency and other biological and/or ecological constraints such as the number of active foragers, soil density, and food availability (Ganeshaiah and Veena 1991; Buhl et al. 2006; Le Comber et al. 2006a, 2006b). Hence, the patterns of the tunnel network may provide an important key to comprehending termite behavior towards maximizing foraging efficiency (Hedlung and Henderson 1999). Lee et al. (2006) showed that termite tunnel pattern has higher efficiency of food encounter rate for randomly distributed food resources, rather than uniform or clumped food distribution. Lee et al. (2007) provided the possible answer to why the length distribution of the branch tunnel follows the exponentially decaying function, *P*(L)∼exp(-α*L*), with a branch length exponent of α = 0.15, where *L* is the branch length of the tunnel. They defined the foraging efficiency as the ratio of energy gain for obtained food to energy loss for transporting food for a given time and showed that when α = 0.15, foraging efficiency was maximized in a landscape. Lee et al. (2008a) and Lee and Su (2009) indirectly showed that termites used two strategies, controlling branching probability and manipulating tunnel growth stopping probability to increase foraging efficiency.

The studies mentioned above were helpful to understand the tunnel network pattern in relation to foraging efficiency. However, they focused on the tunnel network pattern as a whole because it is mathematically difficult to consider the interaction between termite foraging behavior and the local geometry, such as tunnel curvature and roughness. Thus, to overcome this problem, experimental studies are necessary. Lee et al. (2008b) examined termite tunneling in response to tunnel surface irregularity. They revealed that the presence of surface irregularity was essential to induce termite tunneling. Lee et al. (2008c) showed that when termites encountered surface irregularity in the tunnel they responded in either of these ways—the tunneling or non—tunneling behavior—according to the size of the irregularity. Ku et al. (2010) showed that when termites encountered tunnel intersections, they selected a relatively wide tunnel. When considering the speculation that the more frequently used tunnel widths would be wider than less used tunnel widths, and that the wider tunnel would likely be a better candidate for the most efficient path, the selection can be a good strategy for the economic path.

The study by Lee et al. (2008d) contributed to our understanding of the interaction between termite behavior and tunnel geometry on an individual level with the means of movement efficiency. Movement efficiency is the time required for a termite to cover a certain distance in the tunnel. In fact, it can be said that the foraging efficiency consists of two efficiencies, food finding efficiency and food transportation efficiency (Lee et al. 2007). The food finding efficiency is related to tunnel patterns and the transportation efficiency is associated with how quickly termites move their food from the site of food to their nest. Thus, the increase of the movement efficiency can improve the transportation efficiency. In this viewpoint, the movement efficiency is linked to the foraging efficiency. The experimental results showed that termites (*Coptotermes formosanus*) moved faster around smooth—rounded corners than they did around sharp corners, indicating that movement efficiency is higher in the smooth— rounded corners. The smoothness of the tunnel can be mathematically quantified as the curvature. Curvature can be defined as how quickly the slope of a curve is changing about any point along a curve—the higher the curvature, the greater the bending.

In the present study, as a follow—up research to that of Lee et al. (2008d), we measured the time taken by a termite to cover a distance in a tunnel with different curvatures in order to understand how the curvature affects termite traffic efficiency.

## Materials and Methods

The termite *Reticulitermes speratus kyushuensis* (Isoptera: Rhinotermitidae) was collected from monitoring stations by using the method of Su and Scheffrahn (1986). This species is widely distributed in Korea and Japan (Park and Bae 1997). Termites were transported back to the laboratory and separated them from their stations at once, using the methods of Tamashiro et al. (1973). Next, termites were placed inside a release chamber that contained wooden sticks as the food source. The chamber was maintained at a temperature of 26 ± 2 ^°^C.

**Figure 1.**
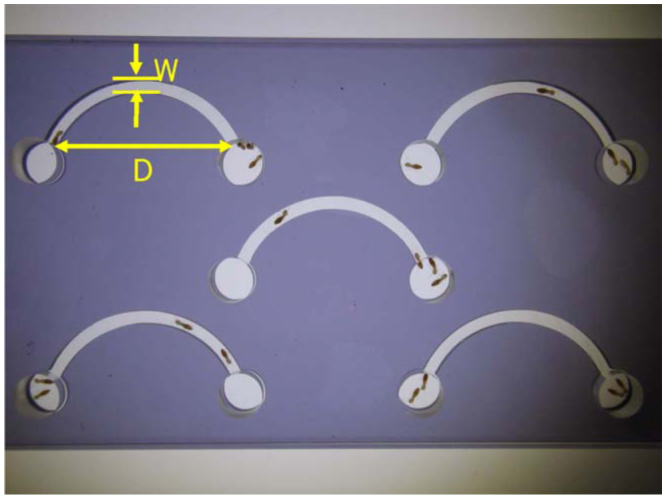
Experimental arena consisting of 2 layers (9 × 16 cm) of clear Plexiglas (thickness, 5 mm) and a middle dark gray— colored layer (9 × 16 cm in size and 2 mm in height). The middle layer with dark gray color has 5 artificial tunnels with a total length of 5 cm (*W* = 3 mm and *D* = 4 cm). High quality figures are available online.

Two—dimensional foraging arenas were used in this study. The experimental arena consisted of three layers (9 × 16 cm) of clear Plexiglas (5 mm thick) with a dark gray— colored middle layer (9 × 16 cm, 2 mm in height) between two outer layers (see Figure 1). Tunnel shapes were cut on the middle layer to be used as an artificial tunnel with varying widths (*W* = 2, 3, or 4 mm) and different distances between the two ends of the tunnel (*D* = 2, 3, 4, or 5 cm). The variable *D* represented the curvature. Mathematically, we define the curvature as a plane curve. The curvature of the curve at a point is a measure of how sensitive its tangent line is to moving the point to other nearby points. Higher curvature means more bending. Thus, the degree of the curvature decreases with the increase of *D*. The ends of the artificial tunnels were connected to the introduction holes (1 cm diameter). The top and middle layers of the arena had holes (1 cm diameter) drilled through them to allow termite entry.

Each arena, containing five artificial tunnels, was placed in a horizontal position in a room kept at 26 ^°^C, and four worker termites were placed into an artificial tunnel through the introduction hole. The four termites acclimatized to their new environment for 10 min. After this period, their movements were recorded for one hour with a digital camcorder mounted above the substrate. Using the recording, the time (τ) taken by a termite to pass through the tunnel was measured. 60 individual tests were performed on each combination of tunnel width and curvature. In the analysis, only worker termites that did not make physical contact with neighboring termites while walking in the arenas were used.

**Figure 2.**
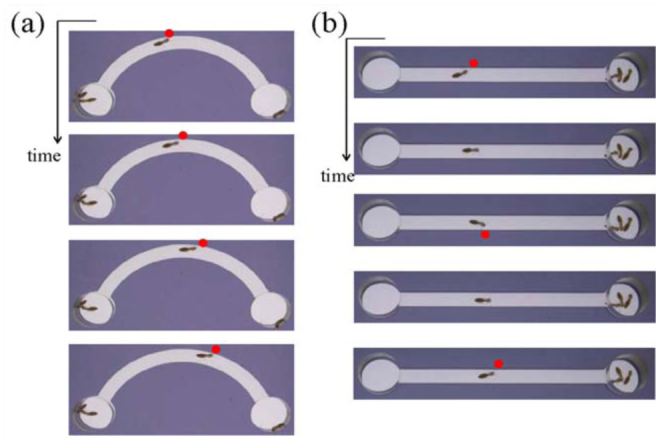
Snapshots of the two types of termite walking behavior in the artificial tunnel: (a) biased walking towards outer wall of the bending tunnel with *W* = 4 mm and *D* = 2 cm, and (b) zigzag walking in a straight tunnel with *W* = 4 mm and *D* = 5 cm. Red circles indicate the position where the termite's antenna touched the wall. High quality figures are available online.

## Results

Termites introduced into the artificial tunnels began to run along the tunnel. When walking through the tunnel, they touched the tunnel walls with their antennae for a short time to obtain information regarding the circumambient geometry.

For *W* = 2 mm, termites required statistically less time to pass through the tunnel with *D* = 5 cm than they did for other *D* values. When termites walked in the tunnel with *D* = 2, 3, or 4 cm, their movement was biased to the outer wall (Figure 2a). The red circle shows the location where the termite's antenna touched the wall. These circles are indicative of biased walking. The biased behavior caused unbalanced antenna touching, frequently causing the termites to hesitate in activity. The hesitation increased the travel time (τ). For *D* = 2, 3, or 4 cm, the effect of the biased behavior on τ was statistically the same because the narrow tunnel width diluted the effect. For *W* = 3 mm, the biased behavior clearly appeared when *D* = 2 or 3 cm. Thus, termites passed slowly through the tunnels with *D* = 2 or 3 cm rather than in those with *D* = 4 or 5 cm. In the cases with *W* = 4 mm, the effect of the biased behavior was pronounced in tunnels with *D* = 2 and 3 cm. Interestingly for *D* = 5 cm, w another factor was observed that increased the travel time. When walking in a wide tunnel, termites walked in a zigzag motion, touching inner and outer walls in a balanced frequency. The zigzag motion led to longer walking distance in comparison to biased walking (Figure 2b). The red circles show the position where the termite's antenna touched the wall. These circles show zigzag walking. Consequently, this behavior caused an increase in τ (see Table 1). In brief, τ increased with an increase in *W* and decreased with an increase in *D* (see Table 1).

## Discussion

Lee et al. (2008c) showed experimentally that termites passed through smooth—rounded corners more quickly than around sharp corners. When termites confront a sharp corner, many stop walking and touch the lateral walls of the tunnel with their antennae. From our study, we infer that travel time is affected by differences in tunnel smoothness. The smoothness can be mathematically quantified as the curvature. The curvature of a curve at a point is a measure of how sensitive its tangent line is to moving the point to other nearby points. Higher curvature means more bending. The experimental results showed that τ decreased with decreasing *W* and increasing *D* (Table 1). This resulted from the two types of termite walking behavior; biased walking towards outer walls in tunnels with smaller values of *D* and balanced zigzag walk in tunnels with larger values of *W.* This means that termites would increase traffic efficiency with the construction of more geometrically straight and narrow tunnels.

**Table 1.**
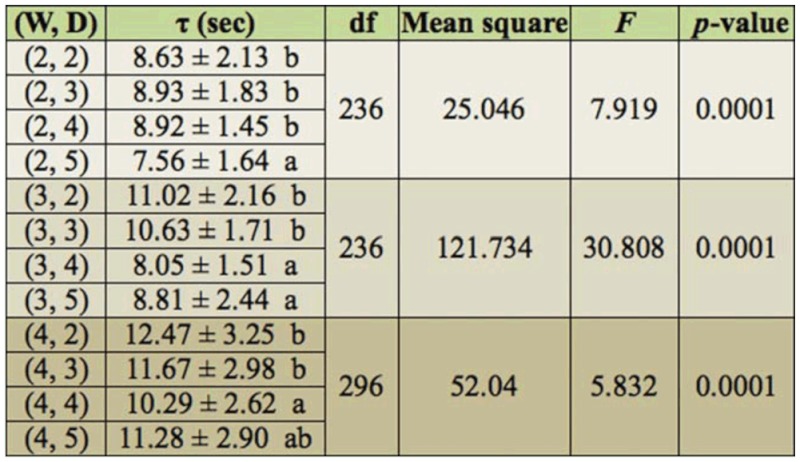
Tunnel passing time, τ (mean ± SD), for different tunnel width, W (mm), and different distances between the 2 ends of the tunnel, D (cm). Sixty individual tests were performed on each combination of W and D.

In the field, curved and/or meandering tunnels are observed. From a Darwinian perspective, termites have evolved towards increasing foraging efficiency, and thus the curved tunnel should be beneficial to foraging efficiency. Termite foraging efficiency is the ratio of obtained energy to consumed energy in a given time (Lee et al. 2007). The obtained energy is a reflection of how effective the termites are at locating food. Consumed energy includes not only the energy expended by the termites while they transport food back to the nest, but also the energy expended when they search for food.

In the theoretical viewpoint, combinational optimization of the two types of energies is most likely to be a solution to understand the reason why termites construct wavy tunnels. However, in the present study, it is difficult to directly discuss the optimization problem, since the experimental results shown here are limited to a single termite's movement. In fact, the situation that many termites experience in a tunnel network is similar to the phenomenon of the pedestrian flow (or traffic behavior) on extensive road networks (May et al. 2008). Thus, it is necessary to investigate traffic efficiency related to transportation behavior at the population level. Additionally, in the field, there could be many other constraints associated with physical factors such as soil hydrology and soil particle size (Su and Puche 2003). Further research is necessary to understand how termites optimize the two types of energies. Nevertheless, our results are valuable in that they provide insights into foraging efficiency at an individual level. These results also suggest directions for future empirical investigations of termite foraging strategy in relation to traffic efficiency.
